# The Association between Health Conditions in World Trade Center Responders and Sleep-Related Quality of Life and Sleep Complaints

**DOI:** 10.3390/ijerph16071229

**Published:** 2019-04-06

**Authors:** Indu Ayappa, Yingfeng Chen, Nisha Bagchi, Haley Sanders, Kathleen Black, Akosua Twumasi, David M. Rapoport, Shou-En Lu, Jag Sunderram

**Affiliations:** 1 Division of Pulmonary, Critical Care and Sleep Medicine Icahn School of Medicine at Mount Sinai, New York, NY 10029, USA; nb2229@nyu.edu (N.B.); haley.sanders@mssm.edu (H.S.); Akosua.Twumasi@mssm.edu (A.T.); david.rapoport@mssm.edu (D.M.R.); 2 School of Public Health, Rutgers University, Piscataway, NJ 08854, USA; yingfengc123@gmail.com (Y.C.); sl1020@sph.rutgers.edu (S.-E.L.); 3 Environmental and Occupational Health Sciences Institute, Rutgers Biomedical and Health Sciences, Piscataway, NJ 08854, USA; kgb3@eohsi.rutgers.edu; 4 Division of Pulmonary and Critical Care Medicine, Robert Wood Johnson Medical School, Rutgers University, New Brunswick, NJ 08901, USA; sunderja@rwjms.rutgers.edu

**Keywords:** obstructive sleep apnea, comorbid insomnia, sleep-related quality of life, chronic sinusitis, sleepiness, WTC responders

## Abstract

*Background:* World Trade Center (WTC) dust-exposed subjects have multiple comorbidities that affect sleep. These include obstructive sleep apnea (OSA), chronic rhinosinusitis (CRS), gastroesophageal-reflux disorder (GERD) and post-traumatic stress disorder (PTSD). We examined the impact of these conditions to sleep-related outcomes. *Methods*: Demographics, co-morbidities and symptoms were obtained from 626 WTC (109F/517M), 33–87years, BMI = 29.96 ± 5.53 kg/m^2^) subjects. OSA diagnosis was from a 2-night home sleep test (ARES^TM^). Subjective sleep quality, sleep-related quality of life (QOL, Functional Outcomes of Sleep Questionnaire), excessive daytime sleepiness (Epworth Sleepiness Scale), sleep duration and sleep onset and maintenance complaints were assessed. *Results*: Poor sleep quality and complaints were reported by 19–70% of subjects and average sleep duration was 6.4 h. 74.8% of subjects had OSA. OSA diagnosis/severity was not associated with any sleep-related outcomes. Sleep duration was lower in subjects with all conditions (*p* < 0.05) except OSA. CRS was a significant risk factor for poor sleep-related QOL, sleepiness, sleep quality and insomnia; PTSD for poor sleep-related QOL and insomnia; GERD for poor sleep quality. These associations remained significant after adjustment for, age, BMI, gender, sleep duration and other comorbidities. *Conclusions*: Sleep complaints are common and related to several health conditions seen in WTC responders. Initial interventions in symptomatic patients with both OSA and comorbid conditions may need to be directed at sleep duration, insomnia or the comorbid condition itself, in combination with intervention for OSA.

## 1. Introduction

Following the World Trade Center (WTC) disaster, many responders who worked in rescue, recovery and debris removal were exposed to high concentrations (greater than 100,000 µm/m^3^ total particles) of debris [1]. This exposure was associated with an increased risk of developing obstructive sleep apnea (OSA) [2], chronic rhinosinusitis (CRS) [3], post-traumatic stress disorder (PTSD), gastroesophageal reflux disease (GERD), anxiety and depression [4] all of which may be associated with sleep disruption. We recently reported a high prevalence of OSA in WTC responders related to chronic rhinosinusitis [5].

OSA is often associated with poor quality of life and sleepiness, likely from the associated sleep fragmentation and intermittent hypoxia. Although there is a weak relationship between presence of OSA and/or severity of OSA and sleep complaints and daytime outcomes, this correlation appears to be quite variable [6,7,8,9,10]. Sleep duration and insomnia may be additional factors affecting the relationship of OSA to sleepiness [6,9]. There is also increasing evidence that medical conditions that often coexist with OSA, such as CRS, anxiety, depression, PTSD and GERD, are associated with sleep complaints [11,12,13,14,15,16,17] and may confound the relationship between OSA and sleep-related quality of life outcomes. The independent contribution of each of these conditions to sleep-related complaints and the associated quality of life has not been closely examined.

Data on sleep-related quality of life, sleepiness and sleep duration were collected as part of a larger study (WTCSNORE) examining the role of nasal pathology in the pathophysiology of OSA in 643 WTC responders. We evaluated the relative contribution of sleep duration and comorbid illness to these outcomes in this population.

## 2. Materials and Methods

Study Population: The WTCSNORE study is a recently completed National Institute for Occupational Safety and Health (NIOSH) funded (#U01OH01415) study to examine the effect of nasal pathology on OSA in WTC responders. Between March 2013 and December 2016 we enrolled 643 subjects with no history of OSA and without reported loud and frequent snoring prior to 11 September 2001 from the WTC health programs at Rutgers Robert Wood Johnson Medical School, New York University School of Medicine and Icahn School Medicine at Mt. Sinai. The study protocol was approved by the Institutional Review Boards of Rutgers RBHS (Pro2012002164), NYU School of Medicine (I12-02578) and the Icahn School of Medicine at Mount Sinai (HS#16-00511) and all subjects signed informed consent. Subjects were considered ineligible if they had been diagnosed with gross skeletal alterations affecting the upper airway (e.g., micrognathia) or unstable chronic medical conditions known to affect OSA (chronic heart failure, stroke), were pregnant or had intent to become pregnant within the period of the protocol, were unable to sign informed consent form, were currently on treatment for OSA, were a habitual snorer prior to 9/11/2001, or had a diagnosis of OSA prior to 9/11/2001. In all subjects we obtained demographic data and the following metrics were assessed:

(1) Sleep-related Quality of life (QOL) was assessed using the Functional Outcomes of Sleep Questionnaire (FOSQ) [18]. The FOSQ is a validated 30-item questionnaire that is used to assess the impact of disorders of excessive sleepiness on activity of daily living. It consists of 5 subscales evaluating activity level, vigilance, intimacy, general productivity and social outcomes and has a possible combined score that ranges from 5 (poor) to 20 (excellent). A score < 17 is taken to indicate poor sleep-related quality of life.
(2) Excessive daytime sleepiness was evaluated with the Epworth Sleep Scale (ESS), a well-validated questionnaire that asks the subject to rate the likelihood of falling asleep in 8 commonly encountered situations [19]. Possible scores range from 0 (the least sleepy) to 24 (the most sleepy). A score of > 10 defines presence of excessive daytime sleepiness.
(3) A questionnaire regarding sleep and snoring was administered that included questions regarding bed and wake up times, duration of sleep (hours/night), frequency of difficulty falling and maintaining sleep per week, and overall quality of sleep. Quality of sleep was rated on a 4-point scale (1: excellent, 2: good, 3: fair, and 4: poor); poor sleep quality was defined as ≥ 3. Sleep onset insomnia was defined as present when the subject reported having trouble falling asleep at least 1–2 times a week, and sleep maintenance insomnia was recorded when a subject reported waking up early and not being able to go back to sleep at least 1–2 days per week.
(4) Home monitoring for OSA: Subjects wore an ARES^TM^ Unicorder (SleepMed, Inc., West Palm Beach, FL, USA) at home for 2 nights, with a pre-addressed mailer to return the device to the sleep lab. The ARES^TM^ Unicorder is a home sleep test device that has been validated against full in-lab polysomnography [20,21], shows high sensitivity for OSA (0.98) and is routinely used in clinical practice for home monitoring of OSA. It is worn on the forehead and does not require additional wires to external devices. It measures oxygen saturation and pulse rate from reflectance oximetry, airflow from a nasal cannula/pressure transducer, snoring via acoustic microphone and head movement actigraphy and head position from accelerometers. The ARES^TM^ respiratory data were analyzed as follows: Data from the monitor were autoscored and then manually edited by a single trained sleep technician and reviewed by a sleep expert. Apneas were scored when there was a reduction in airflow to less than 10% of baseline for >10 s. Hypopneas 4% were scored for >30% reduction in airflow associated with 4% or more decrease in oxygen saturation. Hypopneas with arousals (HypopneasArsl) were scored for visible reduction (>30% reduction) in airflow associated with surrogates of arousal (head movement, changes in snoring, or changes in pulse rate combined with disappearance of inspiratory flow limitation and a marked (>150%) increase in flow amplitude) at the end of the event, but <4% decrease in oxygen saturation [22]. AHI4% was calculated as Apneas+Hypopneas4% divided by total valid recording time in hours (h). The Respiratory Disturbance Index (RDI) was calculated as Apneas + Hypopneas 4% + HypopneasArsl divided by total valid recording time. Using these metrics, we define OSA as present when AHI4% ≥ 5/h or when RDI ≥ 15/h. When OSA is present by this definition, severity was graded by the AHI4%: mild (AHI4% < 15/h), moderate (AHI4% ≥ 15/h but < 30/h) or severe (AHI4% ≥ 30/h) [23].
(5) Chronic rhinosinusitsis (CRS): CRS was defined based on epidemiological criteria used in the European Position Paper on Rhinosinusitis and Nasal Polyps (EPOS) [24,25]. Subjects were considered CRS+ if two or more of the following symptoms were reported as present for >8 weeks: (1) nasal blockade/obstruction (2) nasal discharge, (3) facial pain and (4) reduction in or loss of smell. At least one symptom had to be nasal blockade or discharge.
(6) In addition, we quantified the subjects’ perception of nasal congestion of each individual nostril by asking them to close one nostril while in the supine position and rate the level of congestion in the free nostril on a scale from 1 to 10, 1 being totally blocked/congested and 10 being totally open/uncongested. The lowest score (denoting greater congestion) of the two self-reported values from the left and right nostril was used for analysis.
(7) Comorbid conditions: Information on comorbid Anxiety/Panic, Depression, GERD and PTSD were obtained from a combination of self-report of physician diagnosis (GERD) obtained in our WTCSNORE questionnaire, standardized questionnaires (Depression, Panic disorder, PTSD) and from the certified conditions listed in WTC Health Program General Responder Data Center (GERD, Depression, Anxiety and Panic Disorder and PTSD) for each subject using the annual visit closest in time to the sleep study. If any of the sources used identified the presence of a co-morbid condition the subject was coded as positive for that condition. Depression was assessed by the patient health questionnaire (PHQ), PTSD (PTSD symptom checklist), panic (PHQ) and anxiety from the GAD-7 [26,27,28,29].

Assessment of other relevant variables and Statistical Analysis: Presence/absence of CRS, OSA, PTSD, Anxiety/Depression and GERD were defined as Yes/No (+/−) variables. Summary statistics were calculated and differences were compared using chi-square test for discrete categorical variables and two-sample *t*-test for continuous data. For skewed continuous variables, we used Wilcoxon test instead. Logistic regression analyses, with (i) sleep-related QOL (FOSQ ≥ 17 vs <17), (ii) Excessive Daytime Sleepiness (EDS) (Epworth sleepiness score >10 vs ≤10), (iii) Poor sleep quality (+/−), (iv) Sleep Onset Insomnia (+/−) and (v) and Sleep Maintenance Insomnia (+/−) as the dependent variables and OSA, CRS, GERD, PTSD, Anxiety/Panic and Depression as the independent variables were used to evaluate the association of sleep parameters and each of the independent variables before and after controlling for age, BMI, gender, sleep duration and the other variables. Statistical significance was defined by *p* < 0.05. All statistical analyses were performed using SAS v9.4 (SAS Institute Inc., Cary, NC, USA).

## 3. Results

We analyzed data from 626/643 subjects from the parent WTC study (WTCSNORE) in whom CRS data was available. Subjects were predominantly middle-aged men. The demographic data, sleep duration, sleep-related quality of life and sleep complaints are provided in Table 1. 74.8% of subjects had OSA diagnosed by home monitoring, predominantly mild OSA. 47.1% of the subjects were identified as CRS+ by questionnaire, 19.3% had depression, 23.5% had PTSD, 23.5% had anxiety/panic and 52% had GERD.

Shorter sleep duration (in hours) was observed in subjects with CRS (CRS+ = 6.3 ± 1.4 vs CRS- = 6.5 ± 1.2, *p* = 0.03), PTSD (PTSD+ = 6.1 ± 1.5 vs PTSD- = 6.5 ± 1.3, p=0.01), anxiety/panic (anxiety/panic+ =6.0 ± 1.4 vs anxiety/panic- =6.5 ± 1.3, *p* < 0.0001) and depression (depression+ = 6.0 ± 1.4 vs depression- = 6.5 ± 1.3, *p* = 0.001) but not GERD (GERD+ = 6.4 ± 1.3 vs GERD- = 6.4 ± 1.3, *p* = NS) and OSA (OSA+ = 6.4 ± 1.3 vs OSA- = 6.4 ± 1.3, *p* = NS). Subjects with OSA were older than those without OSA, were predominantly male and had higher BMI, but did not differ in sleep duration, ESS, FOSQ or perception of nasal congestion. Subjects with CRS, PTSD, Anxiety/Panic, Depression and GERD showed greater sleepiness by ESS, worse sleep-related QOL by FOSQ and greater perceived nasal congestion than those without the condition. Of note, subjects with CRS had slightly higher BMI and OSA severity (See Table 2).

Shorter sleep duration was significantly associated with worse sleep-related quality of life (FOSQ < 17, OR = 0.76 95% CI = 0.63, 0.92, *p* = 0.004), worse sleep quality (OR = 0.61, 95% CI 0.50, 0.74, *p* < 0.0001), increased sleepiness (ESS > 10, OR = 0.84, 95% CI 0.72, 0.99, *p* = 0.03), sleep onset insomnia (OR = 0.63, 95% CI 0.53, 0.74, *p* < 0.0001) and maintenance insomnia (OR = 0.54, 95% CI 0.43, 0.67, *p* < 0.0001) using a logistic model controlled for age, BMI, OSA and all comorbidities. In addition, gender was associated with worse sleep-related quality of life (FOSQ) and presence of sleep onset insomnia (*p* < 0.05). Sleep-related quality of life (FOSQ) and sleepiness (ESS) were significantly negatively correlated (*r* = −0.62 95% CI −0.67 to −0.57, *p* < 0.0001) but there was no correlation between sleep disordered breathing indices (SDB, AHI4% or RDI) and FOSQ (*r* = −0.05 for logAHI4% and *r* = −0.01 for logRDI, *p* = NS) or SDB and ESS (*r* = 0.05, for logAHI4%, *r* = 0.03, for logRDI, *p* = NS).

In our dataset, we were unable to demonstrate differences in quality of life, sleepiness, sleep quality, sleep onset insomnia, and sleep maintenance insomnia between subjects with and without OSA. However, presence of the other comorbid conditions (CRS, PTSD, Anxiety/Panic, Depression and GERD) were all significantly associated with worse quality of life, increased sleepiness, worse sleep quality and sleep onset and maintenance insomnia. (Table 3)

**Figure 1:**
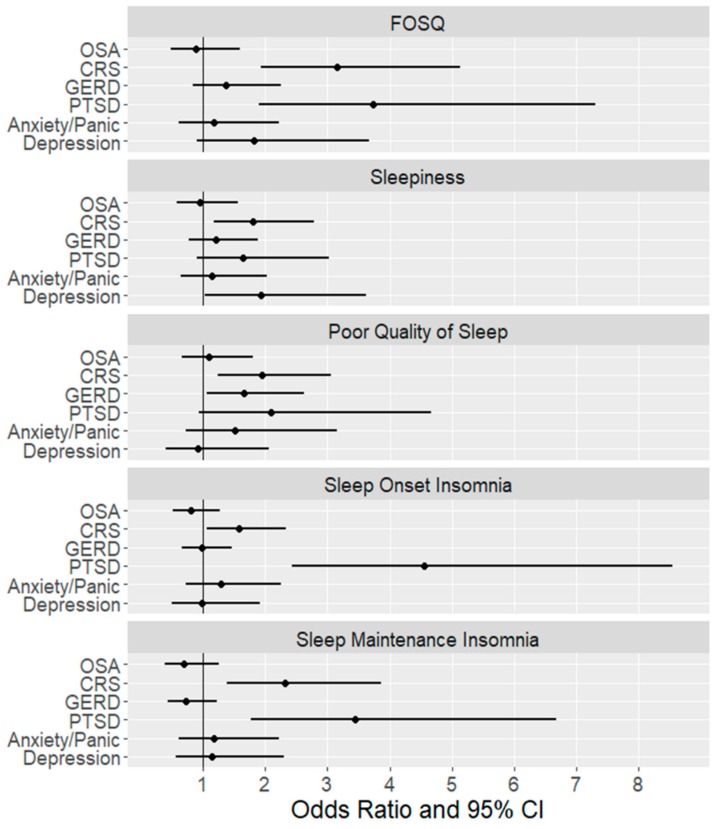
Odds ratio and 95% CI for each comorbid condition as a risk factor for adverse sleep outcomes.

Table 4 summarizes the risk of each of the conditions on sleep-related outcomes and shows the associations of quality of life (FOSQ), excessive daytime sleepiness (ESS), sleep quality, sleep onset insomnia and maintenance insomnia with OSA severity, CRS and other co-morbid conditions. Model 1 is the unadjusted data, Model 2 adjusts for age, BMI and sleep duration and Model 3 adjusts for age, BMI, gender, sleep duration and other co-morbid condition. Our data did not show any statistically significant relationship between OSA and any of the sleep-related outcomes. CRS is a significant risk factor for poor quality of life, excessive daytime sleepiness and sleep complaints even in an adjusted model. PTSD was a significant risk factor for poor quality of life, sleep onset insomnia and sleep maintenance insomnia. GERD was a risk factor for poor quality of sleep. Figure 1 is a graphical representation of these odds ratios in Model 3.

## 4. Discussion

In this large dataset of WTC responders with and without OSA, CRS and other comorbidities that are known to impact sleep and quality of life, we show a significant relationship of sleep-related quality of life, sleepiness and sleep complaints to CRS and PTSD but little or no relationship to the presence of OSA. In patients with OSA, comorbidities that have typically been emphasized include hypertension, arrhythmias, stroke and other cardiovascular diseases [30,31,32]. Our data show that it may also be important to assess other comorbidities that directly impact sleep complaints.

The absence of a relationship between OSA and sleep complaints is not entirely unexpected. While sleepiness is one of the main symptoms of OSA, there is significant variability between individuals and the relationship of OSA (or OSA severity) to quality of life (QOL) and sleepiness has been inconsistent, especially in non-clinical populations [7,8,9,10]. Our data show that controlling for co-morbid conditions did not help explain the variability in OSA and sleep outcomes. Our dataset has a high prevalence of OSA, but the OSA severity (median AHI4% 11.0/h, median RDI 26.0/h in subjects with OSA) was slightly lower than earlier studies of predominantly moderate-severe OSA subjects [33]. However, OSA severity in our cohort is comparable to recent studies in clinical populations [6]—that also did not show a relationship between OSA severity and sleepiness.

Supporting the validity of our self-reported variables, we show the expected relationship between reduced sleep duration and sleepiness and sleep complaints [6]. Average sleep duration has decreased over the recent decades with over 40% of the U.S. population reporting habitual sleep time below the recommended 7–8 h [34]. The association of reduced sleep duration with poor cardiometabolic outcomes [35,36] highlights the importance of our finding of overall short sleep duration in the entire cohort (average 6.4 h with majority having sleep duration <7h) and suggests the need for further investigation and possible intervention.

Our results are also consistent with recent literature showing that individuals with CRS complain of poor sleep and demonstrate poor QOL related to sleep disruption [11,37,38]. However, these studies did not control for OSA severity or sleep duration. We hypothesized that the sleep complaints in CRS could be due to an increase in OSA, but instead found only a relationship to reduced sleep duration. Subjects with CRS in our study reported greater perception of nasal congestion when awake, suggesting that discomfort from congestion during sleep results in the reduced sleep duration and may account for this finding. However, the relationship of CRS to poor sleep remained even after controlling for sleep duration. We have shown a relationship of CRS to level of WTC exposure [5], and chronic inflammation has also been shown to be part of CRS; it is possible that systemic mediators of inflammation such as cytokines contribute to sleep disruption [37,39].

Poor sleep and sleepiness have also been shown in patients with mental health conditions like PTSD, anxiety and depression [8,40,41]. In our dataset all these conditions were highly correlated with one another, but also correlated with sleep complaints.

Although the relationship of sleep related outcomes to comorbid conditions remained significant despite controlling for BMI in the models, subjects with each of the conditions had a higher BMI than those without (see Table 2). Obesity is associated with short sleep duration [42] and may impact the sleep-related complaints in these subjects.

Strengths of our study include the large well-characterized dataset of subjects naïve to OSA treatment, objective measurement of OSA in all subjects, concurrent assessment of sleep complaints and data on confounders and comorbid conditions.

One potential limitation is that the incidence of both OSA and comorbid conditions was high in our cohort that is comprised mainly of male subjects. However, it has been demonstrated that the severity and type (obstructive vs central) of the OSA in the WTC responders was not different from a general clinic population, suggesting generalizability of our findings, of the correlations and of our causal inferences [43]. Furthermore, other specific groups such as veterans, the elderly [44,45] and some general populations have been shown to have a similarly high prevalence of OSA and these co-morbid conditions, and we suggest that our inferences may apply to these groups also. Another potential limitation is that while OSA was objectively assessed in all subjects, all other variables were based on a combination of self-report diagnoses, questionnaire and certification data from the general WTC database with possibility of being overestimated. However, our prevalence estimates are similar to those reported in WTC rescue and recovery workers [46].

## 5. Conclusions

Our data highlight the high prevalence of sleep-related complaints and poor quality of sleep in WTC responders. Our results challenge the often accepted clinical practice that in a patient with sleep complaints, the most urgent treatment needs to be directed to any OSA found. While OSA undoubtedly produces sleep symptoms in some, the role of other frequently found comorbid conditions, including CRS, PTSD, Anxiety/Panic, Depression and GERD, should not be underestimated and perhaps pursued more aggressively earlier. Initial interventions in symptomatic patients with both OSA and comorbid conditions may need to be directed at sleep duration, insomnia or the comorbid condition in combination with intervention for OSA.

## Figures and Tables

**Table 1:** Demographics of the study participants.

Variable	*N* of Valid Data	Summary
Age (years, Mean ± SD)	626	52.8 ± 8.6
BMI (kg/m^2^, Mean ± SD)	626	29.9 ± 5.5
Female (%)	626	109 (17.3%)
Sleep Duration (h, Mean ± SD) ≥7 h (%) 6–6.99 h (%) <6 h (%)	588	6.4 ± 1.3 42.6% 30.9% 26.5%
Snoring (Yes, %)	626	312 (49.8%)
Quality of Life (FOSQ) (Mean ± SD) Good, ≥17 (%) Poor, <17 (%)	566	17.4 ± 2.6 62.0% 38.0%
Sleepiness (ESS, Mean ± SD) Sleepy, >10 (%) Not Sleepy, ≤10 (%)	620	8.3 ± 4.8 31.3% 68.7%
Poor Sleep Quality (Yes, %)	623	441 (70.8%)
Sleep Onset Insomnia (Yes, %)	609	296 (48.6%)
Sleep Maintenance Insomnia (Yes, %)	622	116 (18.7%)
OSA + (%) Mild Moderate Severe	592	443 (74.8%) 274 (46.3%) 112 (18.9%) 57 (9.6%)
CRS + (%)	626	295 (47.1%)
Depression + (%)	612	118 (19.3%)
PTSD + (%)	612	144 (23.5%)
Anxiety and Panic Disorder + (%)	612	144 (23.5%)
GERD + (%)	564	293 (52%)

Notes: BMI-Body Mass Index, ESS- Epworth sleepiness scores, FOSQ- Functional Outcome of Sleep Questionnaire, OSA-Obstructive Sleep Apnea, PSTD-Post Traumatic Stress Disorder, GERD- Gastroesophageal Reflux Disease.

**Table 2:** Comparison of demographic and sleep related variables in subjects with and without OSA, CRS, GERD, PTSD and Anxiety/Panic and Depression.

		Age	Female	BMI kg/m^2^	Sleep Duration (h)	Sleepiness (ESS)	Quality of Life (FOSQ)	AHI4%/h	RDI/h	Perception of Nasal Congestion at time of Visit
		Mean ± SD	N (%)	Mean ± SD	Mean ± SD	Mean ± SD	Mean ± SD	Median (Q1, Q3)	Median (Q1, Q3)	Mean ± SD
OSA+ OSA-	*N* = 443 *N* = 149	53.5 ± 8.3 ^‡^ 50.3 ± 8.8	58 (13.1%) ^‡^ 44 (29.5%)	30.7 ± 5.5 ^‡^ 27.5 ± 4.8	6.4 ± 1.3 6.4 ± 1.3	8.4 ± 4.9 8.0 ± 4.5	17.4 ± 2.7 17.6 ± 2.5	11 (6, 19) ^‡^ 2 (1, 3)	26 (18, 37) ^‡^ 9 (8, 12)	6.8 ± 2.2 6.8 ± 2.2
CRS+ CRS-	*N* = 295 *N* = 331	52.9 ± 8.8 52.7 ± 8.4	46 (15.7%) 46 (18.8%)	30.3 ± 5.3 * 29.6 ± 5.7	6.3 ± 1.4 * 6.5 ± 1.2	9.2 ± 5.1 ^‡^ 7.4 ± 4.5	16.7 ± 3.0 ^‡^ 18.1 ± 2.1	9 (4, 17) * 6 (3, 15)	22 (15, 35) 19 (12, 32)	6.2 ± 2.2 ^‡^ 7.3 ± 2.1
GERD+ GERD-	*N* = 293 *N* = 271	52.6 ± 8.0 52.9 ± 9.2	45 (15.4%) 54 (20%)	30.9 ± 6.1 ^‡^ 28.8 ± 4.9	6.4 ± 1.3 6.4 ± 1.3	9.0 ± 5.0 ^Ɨ^ 7.7 ± 4.7	16.9 ± 2.9 ^‡^ 18.0 ± 2.3	8 (4, 17) 7 (3, 14.5)	22 (15, 34) 20 (12, 32)	6.3 ± 2.2 ^‡^ 7.4 ± 2.0
PTSD+ PTSD-	*N* = 144 *N* = 468	51.8 ± 8.0 53.1 ± 8.8	25 (17.4%) 82 (17.5%)	31.3 ± 6.2 ^Ɨ^ 29.5 ± 5.3	6.1 ± 1.5 * 6.5 ± 1.3	10.8 ± 5.3 ^‡^ 7.5 ± 4.4	15.3 ± 3.4 ^‡^ 18.1 ± 2.0	8 (4, 16) 7 (3, 16)	23 (14, 35) 20 (13, 33)	6.0 ± 2.2 ^‡^ 7.0 ± 2.2
Anxiety/Panic+ Anxiety/Panic-	*N* = 144 *N* = 468	51.9 ± 8.3 53.1 ± 8.7	24 (16.7%) 83 (17.7%)	30.9 ± 5.8 * 29.6 ± 5.4	6.0 ± 1.4 ^‡^ 6.5 ± 1.3	10.6 ± 5.2 ^‡^ 7.5 ± 4.4	15.6 ± 3.4 ^‡^ 18.0 ± 2.1	8 (4, 17) 7 (3, 15)	21 (14, 33) 20 (13, 33)	6.2 ± 2.3 ^Ɨ^ 7.0 ± 2.2
Depression+ Depression-	*N* = 118 *N* = 494	52.9 ± 8.3 52.8 ± 8.7	22 (18.6%) 85 (17.2%)	31.5 ± 6.8 * 29.5 ± 5.1	6.0 ± 1.4 ^Ɨ^ 6.5 ± 1.3	10.7 ± 5.4 ^‡^ 7.7 ± 4.5	15.4 ± 3.4 ^‡^ 17.9 ± 2.2	8 (4, 15) 7 (3, 16)	21 (13, 33) 20 (14, 33)	5.9 ± 2.3 ^‡^ 7.0 ± 2.1

Notes: OSA-Obstructive Sleep Apnea, PSTD-Post Traumatic Stress Disorder, GERD- Gastroesophageal Reflux Disease CRS- Chronic Rhinosinusitis. BMI-Body Mass Index, h-hour. Asterisk (*) denotes a *p*-value of <0.05. Cross (Ɨ) denotes a *p*-value of <0.01. Double dagger (‡) denotes a *p*-value of <0.0001.

**Table 3:** Proportion of subjects with sleep related complaints in subjects with and without each condition.

		Poor Sleep-Related Quality of Life	Sleepiness	Poor Sleep Quality	Sleep Onset Insomnia	Sleep Maintenance Insomnia
		FOSQ < 17	ESS > 10	Yes	Present	Present
**OSA+** **OSA-**	*N* = 443 *N* = 149	172 (38.8%) 52 (34.9%)	136 (31.0%) 45 (30.2%)	315 (71.3%) 99 (66.4%)	207 (48.0%) 71 (48.6%)	81 (18.3%) 25 (16.9%)
**CRS +** **CRS-**	*N* = 295 *N* = 331	146 (49.5%) ^‡^ 91 (27.5%)	116 (39.9%) ^‡^ 77 (23.5%)	232 (79.5%) ^‡^ 208 (63.0%)	162 (56.8%) ^‡^ 133 (41.2%)	76 (26.0%) ^‡^ 40 (12.12%)
**GERD+** **GERD-**	*N* = 293 *N* = 271	132 (45.0%) ^Ɨ^ 85 (31.4%)	111 (37.9%) ^Ɨ^ 67 (25.1%)	231 (78.8%) ^‡^ 171 (63.3%)	153 (53.5%) ^Ɨ^ 114 (43.2%)	60 (20.6%) 45 (16.7%)
**PTSD+** **PTSD-**	*N* = 144 *N* = 468	92 (63.9%) ^‡^ 138 (29.5%)	73 (51.0%) ^‡^ 117 (25.2%)	123 (85.4%) ^‡^ 310 (66.4%)	110 (78.0%) ^‡^ 184 (40.4%)	52 (36.1%) ^‡^ 62 (13.3%)
**Anxiety/Panic+** **Anxiety/Panic-**	*N* = 144 *N* = 468	85 (59.0%) ^‡^ 145 (31.0%)	68 (47.6%) ^‡^ 122 (26.2%)	123 (86.0%) ^‡^ 310 (66.2%)	93 (66.4%) ^‡^ 201 (44.0%)	47 (32.6%) ^‡^ 67 (14.4%)
**Depression+** **Depression-**	*N* = 118 *N* = 494	76 (64.4%) ^‡^ 154 (31.2%)	61 (52.1%) ^‡^ 129 (26.3%)	98 (83.8%) ^Ɨ^ 335 (67.8%)	83 (72.8%) ^‡^ 211 (43.7%)	39 (33.0%) ^‡^ 75 (15.2%)

Notes: Asterisk (*) denotes a *p*-value of <0.05. Cross (Ɨ) denotes a *p*-value of <0.01. Double dagger (‡) denotes a *p*-value of <0.0001.

**Table 4:** Odds ratio and 95% CI for presence of poor sleep-related quality of life (FOSQ < 17), sleepiness, poor quality sleep and insomnia in subjects with each condition compared to subjects without this condition.

		Model 1		Model 2		Model 3	
		OR ^1^	95% CI	OR ^2^	95% CI	OR ^3^	95% CI
Poor Quality of Life (FOSQ < 17)	OSA Severity						
	Mild OSA vs. No OSA	1.21	(0.80, 1.84)	1.02	(0.65, 1.59)	0.87	(0.51, 1.47)
	Moderate OSA vs. No OSA	1	(0.60, 1.67)	0.8	(0.46, 1.41)	0.73	(0.36, 1.45)
	Severe OSA vs. No OSA	1.46	(0.78, 2.72)	0.98	(0.50,1.95)	1.09	(0.48, 2.49)
	CRS	2.58 ^‡^	(1.85, 3.60)	2.44 ^‡^	(1.72, 3.46)	2.48 ^‡^	(1.62, 3.80)
	GERD	1.79 ^Ɨ^	(1.27, 2.53)	1.80 ^Ɨ^	(1.24, 2.60)	1.14	(0.74, 1.76)
	PTSD	4.23 ^‡^	(2.85, 6.27)	3.93 ^‡^	(2.60, 5.94)	3.18 ^Ɨ^	(1.72, 5.9)
	Anxiety/Panic	3.21 ^‡^	(2.18, 4.72)	2.87 ^‡^	(1.91, 4.29)	1.18	(0.66, 2.12)
	Depression	3.99 ^‡^	(2.62, 6.09)	3.61 ^‡^	(2.33, 5.59)	1.62	(0.85, 3.11)
Sleepiness (ESS>10)	OSA Severity						
	Mild OSA vs. No OSA	1	(0.65, 1.54)	1.07	(0.67, 1.71)	0.9	(0.53, 1.52)
	Moderate OSA vs. No OSA	1.08	(0.63,1.84)	1.2	(0.67, 2.15)	1.07	(0.55, 2.08)
	Severe OSA vs. No OSA	1.16	(0.60, 2.22)	1.09	(0.53, 2.24)	0.97	(0.42, 2.22)
	CRS	2.16 ^‡^	(1.53,3.06)	1.93 ^Ɨ^	(1.34, 2.77)	1.80 ^Ɨ^	(1.17, 2.76)
	GERD	1.82 ^Ɨ^	(1.27, 2.62)	1.73 ^Ɨ^	(1.18, 2.55)	1.2	(0.78, 1.87)
	PTSD	3.10 ^‡^	(2.10, 4.58)	2.71 ^‡^	(1.80, 4.09)	1.64	(0.89, 3.02)
	Anxiety/Panic	2.55 ^‡^	(1.73, 3.76)	2.03 ^Ɨ^	(1.35, 3.06)	1.12	(0.63, 2.01)
	Depression	3.06 ^‡^	(2.02, 4.63)	2.62 ^‡^	(1.70, 4.05)	1.99	(1.05, 3.76)
Poor Sleep Quality	OSA Severity						
	Mild OSA vs. No OSA	1.16	(0.75, 1.77)	1.21	(0.75, 1.94)	1.05	(0.62, 1.78)
	Moderate OSA vs. No OSA	1.38	(0.81,2.37)	1.28	(0.70, 2.36)	1.04	(0.51, 2.13)
	Severe OSA vs. No OSA	1.55	(0.78,3.10)	1.39	(0.64, 3.04)	1.31	(0.55, 3.15)
	CRS	2.27 ^‡^	(1.58,3.25)	2.20 ^‡^	(1.49,3.25)	1.95 ^Ɨ^	(1.24, 3.07)
	GERD	2.16 ^‡^	(1.48,3.14)	2.21 ^‡^	(1.46, 3.33)	1.67 *	(1.06, 2.64)
	PTSD	2.97 ^‡^	(1.80,4.90)	2.45 ^Ɨ^	(1.44, 4.16)	2.13	(0.96, 4.76)
	Anxiety/Panic	3.13 ^‡^	(1.88,5.22)	2.38 ^Ɨ^	(1.40, 4.06)	1.49	(0.71, 3.13)
	Depression	2.45 ^Ɨ^	(1.45, 4.14)	1.96 *	(1.13, 3.42)	0.95	(0.42, 2.14)
Sleep Onset Insomnia	OSA Severity						
	Mild OSA vs. No OSA	0.89	(0.59, 1.33)	0.79	(0.52, 1.20)	0.85	(0.53, 1.38)
	Moderate OSA vs. No OSA	1.13	(0.69,1.86)	0.94	(0.56, 1.57)	0.96	(0.51, 1.79)
	Severe OSA vs. No OSA	1.1	(0.59,2.04)	0.83	(0.43, 1.61)	1.05	(0.49, 2.24)
	CRS	1.88 ^‡^	(1.36, 2.60)	1.85 ^Ɨ^	(1.34, 2.55)	1.63 *	(1.10, 2.41)
	GERD	1.51 *	(1.08, 2.12)	1.44 *	(1.02, 2.02)	0.99	(0.66, 1.47)
	PTSD	5.25 ^‡^	(3.38, 8.15)	5.22 ^‡^	(3.34, 8.14)	4.69 ^‡^	(2.49, 8.86)
	Anxiety/Panic	2.52 ^‡^	(1.70, 3.75)	2.48 ^‡^	(1.66, 3.70)	1.26	(0.71, 2.23)
	Depression	3.45 ^‡^	(2.20, 5.41)	3.31 ^‡^	(2.10, 5.20)	0.98	(0.50, 1.92)
Sleep Maintenance Insomnia	OSA Severity						
	Mild OSA vs. No OSA	1.1	(0.65, 1.87)	0.93	(0.54, 1.60)	0.85	(0.46, 1.56)
	Moderate OSA vs. No OSA	1.07	(0.56, 2.04)	0.8	(0.41, 1.58)	0.56	(0.25, 1.26)
	Severe OSA vs. No OSA	1.18	(0.54, 2.58)	0.78	(0.34, 1.80)	0.75	(0.29, 1.95)
	CRS	2.54 ^‡^	(1.67, 3.87)	2.49 ^‡^	(1.63, 3.81)	2.41 ^Ɨ^	(1.44, 4.04)
	GERD	1.29	(0.84, 1.98)	1.2	(0.77, 1.85)	0.74	(0.44, 1.24)
	PTSD	3.68 ^‡^	(2.39, 5.68)	3.62 ^‡^	(2.33, 5.63)	3.53 ^‡^	(1.82, 6.88)
	Anxiety/Panic	2.89 ^‡^	(1.87, 4.45)	2.85 ^‡^	(1.83, 4.42)	1.24	(0.64, 2.37)
	Depression	2.74 ^‡^	(1.74, 4.33)	2.57 ^‡^	(1.62, 4.09)	1.06	(0.52, 2.16)

Notes: Asterisk (*) denotes a *p*-value of <0.05. Cross (Ɨ) denotes a *p*-value of <0.01. Double dagger (‡) denotes a *p*-value of <0.0001; 1: Unadjusted odds ratio estimates. 2: For responses of “Poor quality of sleep”, “Sleepy” and “FOSQ”, the odds ratio estimates are adjusted for age, BMI and sleep duration; for responses of “Sleep onset Insomnia” and “Sleep Maintenance Insomnia”, the odds ratio estimates are adjusted for age and BMI. 3 For responses of “Poor quality of sleep”, “Sleepy” and “FOSQ”, the odds ratio estimates are adjusted for Age, BMI, gender, sleep duration, and all other comorbidities; for response “Sleep onset Insomnia” and “Sleep Maintenance Insomnia”, the odds ratio estimates are adjusted for age, BMI, gender and all other comorbidities.

## Abbreviations

OSA	Obstructive Sleep Apnea
BMI	Body Mass Index
H	Hour/s
CI	Confidence Interval
OR	Odds Ratio
CRS	Chronic Rhinosinusitis
GERD	Gastroesophageal Reflux Disorder
PTSD	Post Traumatic Stress Disorder
WTC	World Trade Center
FOSQ	Functional Outcomes of Sleep Questionnaire
QOL	Quality of Life
AHI4%	Apnea Hypopnea (with 4% O2 desaturation) Index
RDI	Respiratory Disturbance Index
SDB	Sleep Disordered Breathing
PAP	Positive Airway Pressure

## References

[1] Lioy P.J., Georgopoulos P. (2006). The anatomy of the exposures that occurred around the World Trade Center site: 9/11 and beyond. Ann. N. Y. Acad. Sci..

[2] Ahuja S., Zhu Z., Shao Y., Berger K.I., Reibman J., Ahmed O. (2018). Obstructive Sleep Apnea in Community Members Exposed to World Trade Center Dust and Fumes. J. Clin. Sleep Med..

[3] Weakley J., Hall C.B., Liu X., Zeig-Owens R., Webber M.P., Schwartz T., Prezant D. (2016). The effect of World Trade Center exposure on the latency of chronic rhinosinusitis diagnoses in New York City firefighters: 2001–2011. Occup. Environ. Med..

[4] Bowler R.M., Kornblith E.S., Li J., Adams S.W., Gocheva V.V., Schwarzer R., Cone J.E. (2016). Police officers who responded to 9/11: Comorbidity of PTSD, depression, and anxiety 10–11 years later. Am. J. Ind. Med..

[5] Sunderram J., Weintraub M., Black K., Alimokhtari S., Twumasi A., Sanders H., Udasin I., Harrison D., Chitkara N., de la Hoz R.E. (2019). Chronic Rhinosinusitis Is an Independent Risk Factor for OSA in World Trade Center Responders. Chest.

[6] Prasad B., Steffen A.D., Van Dongen H.P.A., Pack F.M., Strakovsky I., Staley B., Dinges D.F., Maislin G., Pack A.I., Weaver T.E. (2018). Determinants of sleepiness in obstructive sleep apnea. Sleep.

[7] Budhiraja R., Kushida C.A., Nichols D.A., Walsh J.K., Simon R.D., Gottlieb D.J., Quan S.F. (2017). Predictors of sleepiness in obstructive sleep apnoea at baseline and after 6 months of continuous positive airway pressure therapy. Eur. Respir. J..

[8] Bixler E.O., Vgontzas A.N., Lin H.M., Calhoun S.L., Vela-Bueno A., Kales A. (2005). Excessive daytime sleepiness in a general population sample: The role of sleep apnea, age, obesity, diabetes, and depression. J. Clin. Endocrinol. Metab..

[9] Kapur V.K., Baldwin C.M., Resnick H.E., Gottlieb D.J., Nieto F.J. (2005). Sleepiness in patients with moderate to severe sleep-disordered breathing. Sleep.

[10] Gottlieb D.J., Whitney C.W., Bonekat W.H., Iber C., James G.D., Lebowitz M., Nieto F.J., Rosenberg C.E. (1999). Relation of sleepiness to respiratory disturbance index: The Sleep Heart Health Study. Am. J. Respir. Crit. Care Med..

[11] Bengtsson C., Lindberg E., Jonsson L., Holmstrom M., Sundbom F., Hedner J., Malinovschi A., Middelveld R., Forsberg B., Janson C. (2017). Chronic Rhinosinusitis Impairs Sleep Quality: Results of the GA2LEN Study. Sleep.

[12] Gregory A.M., Buysse D.J., Willis T.A., Rijsdijk F.V., Maughan B., Rowe R., Cartwright S., Barclay N.L., Eley T.C. (2011). Associations between sleep quality and anxiety and depression symptoms in a sample of young adult twins and siblings. J. Psychosom. Res..

[13] Westermeyer J., Khawaja I., Freerks M., Sutherland R.J., Engle K., Johnson D., Thuras P., Rossom R., Hurwitz T. (2010). Correlates of daytime sleepiness in patients with posttraumatic stress disorder and sleep disturbance. Prim. Care Companion J. Clin. Psychiatry.

[14] Lindam A., Jansson C., Nordenstedt H., Pedersen N.L., Lagergren J. (2012). A population-based study of gastroesophageal reflux disease and sleep problems in elderly twins. PLoS ONE.

[15] Zheng M., Wang X., Ge S., Gu Y., Ding X., Zhang Y., Ye J., Zhang L. (2017). Allergic and Non-Allergic Rhinitis Are Common in Obstructive Sleep Apnea but Not Associated With Disease Severity. J. Clin. Sleep Med..

[16] Pan M.L., Tsao H.M., Hsu C.C., Wu K.M., Hsu T.S., Wu Y.T., Hu G.C. (2016). Bidirectional association between obstructive sleep apnea and depression: A population-based longitudinal study. Medicine (Baltim.).

[17] You C.R., Oh J.H., Seo M., Lee H.Y., Joo H., Jung S.H., Lee S.H., Choi M.G. (2014). Association Between Non-erosive Reflux Disease and High Risk of Obstructive Sleep Apnea in Korean Population. J. Neurogastroenterol. Motil..

[18] Weaver T.E., Laizner A.M., Evans L.K., Maislin G., Chugh D.K., Lyon K., Smith P.L., Schwartz A.R., Redline S., Pack A.I. (1997). An instrument to measure functional status outcomes for disorders of excessive sleepiness. Sleep.

[19] Johns M.W. (1991). A new method for measuring daytime sleepiness: The Epworth sleepiness scale. Sleep.

[20] Ayappa I., Norman R.G., Seelall V., Rapoport D.M. (2008). Validation of a self-applied unattended monitor for sleep disordered breathing. J. Clin. Sleep Med..

[21] Westbrook P.R., Levendowski D.J., Cvetinovic M., Zavora T., Velimirovic V., Henninger D., Nicholson D. (2005). Description and validation of the apnea risk evaluation system: A novel method to diagnose sleep apnea-hypopnea in the home. Chest.

[22] Ayappa I., Norman R.G., Krieger A.C., Rosen A., O’Malley R L., Rapoport D.M. (2000). Non-Invasive detection of respiratory effort-related arousals (REras) by a nasal cannula/pressure transducer system. Sleep.

[23] American Academy of Sleep Medicine Task Force (1999). Sleep-related breathing disorders in adults: Recommendations for syndrome definition and measurement techniques in clinical research. The Report of an American Academy of Sleep Medicine Task Force. Sleep.

[24] Fokkens W.J., Lund V.J., Mullol J., Bachert C., Alobid I., Baroody F., Cohen N., Cervin A., Douglas R., Gevaert P. (2012). European Position Paper on Rhinosinusitis and Nasal Polyps 2012. Rhinol. Suppl..

[25] Fokkens W.J., Lund V.J., Mullol J., Bachert C., Alobid I., Baroody F., Cohen N., Cervin A., Douglas R., Gevaert P. (2012). EPOS 2012: European position paper on rhinosinusitis and nasal polyps 2012. A summary for otorhinolaryngologists. Rhinology.

[26] Kroenke K., Spitzer R.L., Williams J.B. (2001). The PHQ-9: Validity of a brief depression severity measure. J. Gen. Intern. Med..

[27] Spitzer R.L., Kroenke K., Williams J.B. (1999). Validation and utility of a self-report version of PRIME-MD: The PHQ primary care study. Primary Care Evaluation of Mental Disorders. Patient Health Questionnaire. JAMA.

[28] Spitzer R.L., Kroenke K., Williams J.B., Lowe B. (2006). A brief measure for assessing generalized anxiety disorder: The GAD-7. Arch. Intern. Med..

[29] Blanchard E.B., Jones-Alexander J., Buckley T.C., Forneris C.A. (1996). Psychometric properties of the PTSD Checklist (PCL). Behav. Res. Ther..

[30] Peppard P.E., Young T., Palta M., Skatrud J. (2000). Prospective study of the association between sleep-disordered breathing and hypertension. N. Engl. J. Med..

[31] Yaggi H.K., Concato J., Kernan W.N., Lichtman J.H., Brass L.M., Mohsenin V. (2005). Obstructive sleep apnea as a risk factor for stroke and death. N. Engl. J. Med..

[32] Mehra R., Benjamin E.J., Shahar E., Gottlieb D.J., Nawabit R., Kirchner H.L., Sahadevan J., Redline S. (2006). Association of nocturnal arrhythmias with sleep-disordered breathing: The Sleep Heart Health Study. Am. J. Respir. Crit. Care Med..

[33] Quan S.F., Chan C.S., Dement W.C., Gevins A., Goodwin J.L., Gottlieb D.J., Green S., Guilleminault C., Hirshkowitz M., Hyde P.R. (2011). The association between obstructive sleep apnea and neurocognitive performance—The Apnea Positive Pressure Long-term Efficacy Study (APPLES). Sleep.

[34] Hirshkowitz M., Whiton K., Albert S.M., Alessi C., Bruni O., DonCarlos L., Hazen N., Herman J., Adams Hillard P.J., Katz E.S. (2015). National Sleep Foundation’s updated sleep duration recommendations: Final report. Sleep Health.

[35] St-Onge M.P., Grandner M.A., Brown D., Conroy M.B., Jean-Louis G., Coons M., Bhatt D.L. (2016). Sleep Duration and Quality: Impact on Lifestyle Behaviors and Cardiometabolic Health: A Scientific Statement From the American Heart Association. Circulation.

[36] Cappuccio F.P., Cooper D., D’Elia L., Strazzullo P., Miller M.A. (2011). Sleep duration predicts cardiovascular outcomes: A systematic review and meta-analysis of prospective studies. Eur. Heart J..

[37] Alt J.A., Smith T.L. (2013). Chronic rhinosinusitis and sleep: A contemporary review. Int. Forum Allergy Rhinol..

[38] Alt J.A., Smith T.L., Mace J.C., Soler Z.M. (2013). Sleep quality and disease severity in patients with chronic rhinosinusitis. Laryngoscope.

[39] Mahdavinia M., Schleimer R.P., Keshavarzian A. (2017). Sleep disruption in chronic rhinosinusitis. Expert Rev. Anti-Infect. Ther..

[40] Ohayon M.M., Shapiro C.M. (2000). Sleep disturbances and psychiatric disorders associated with posttraumatic stress disorder in the general population. Compr. Psychiatry.

[41] Jenkins M.M., Colvonen P.J., Norman S.B., Afari N., Allard C.B., Drummond S.P. (2015). Prevalence and Mental Health Correlates of Insomnia in First-Encounter Veterans with and without Military Sexual Trauma. Sleep.

[42] Cappuccio F.P., Taggart F.M., Kandala N.B., Currie A., Peile E., Stranges S., Miller M.A. (2008). Meta-analysis of short sleep duration and obesity in children and adults. Sleep.

[43] de la Hoz R.E., Aurora R.N., Landsbergis P., Bienenfeld L.A., Afilaka A.A., Herbert R. (2010). Snoring and obstructive sleep apnea among former World Trade Center rescue workers and volunteers. J. Occup. Environ. Med..

[44] Haba-Rubio J., Marques-Vidal P., Andries D., Tobback N., Preisig M., Vollenweider P., Waeber G., Luca G., Tafti M., Heinzer R. (2015). Objective sleep structure and cardiovascular risk factors in the general population: The HypnoLaus Study. Sleep.

[45] Heinzer R., Vat S., Marques-Vidal P., Marti-Soler H., Andries D., Tobback N., Mooser V., Preisig M., Malhotra A., Waeber G. (2015). Prevalence of sleep-disordered breathing in the general population: The HypnoLaus study. Lancet Respir. Med..

[46] Wisnivesky J.P., Teitelbaum S.L., Todd A.C., Boffetta P., Crane M., Crowley L., de la Hoz R.E., Dellenbaugh C., Harrison D., Herbert R. (2011). Persistence of multiple illnesses in World Trade Center rescue and recovery workers: A cohort study. Lancet.

